# Arid3c identifies an uncharacterized subpopulation of V2 interneurons during embryonic spinal cord development

**DOI:** 10.3389/fncel.2024.1466056

**Published:** 2024-10-16

**Authors:** Estelle Renaux, Charlotte Baudouin, Olivier Schakman, Ondine Gay, Manon Martin, Damien Marchese, Younès Achouri, René Rezsohazy, Françoise Gofflot, Frédéric Clotman

**Affiliations:** ^1^Université catholique de Louvain, Louvain Institute of Biomolecular Science and Technology, Animal Molecular and Cellular Biology group, Louvain-la-Neuve, Belgium; ^2^Université catholique de Louvain, Institute of Neuroscience, Laboratory of Neural Differentiation, Brussels, Belgium; ^3^Université catholique de Louvain, Institute of Neuroscience, Behavioral Analysis Platform (BEAP), Brussels, Belgium; ^4^Master de Biologie, École Normale Supérieure de Lyon, Université Claude Bernard Lyon 1, Université de Lyon, Lyon Cedex, France; ^5^Université catholique de Louvain, Louvain Institute of Biomolecular Science and Technology, Louvain-la-Neuve, Belgium; ^6^Université catholique de Louvain, de Duve Institute, Transgenic Core Facility, Brussels, Belgium

**Keywords:** embryonic spinal cord, V2 interneurons, V2c interneurons, Arid3c, motor circuits, motor activity

## Abstract

Motor activity is organized by neuronal networks composed of motor neurons and a wide variety of pre-motor interneuron populations located in the brainstem and spinal cord. Differential expression and single-cell RNA sequencing studies recently unveiled that these populations subdivide into multiple subsets. However, some interneuron subsets have not been described yet, and the mechanisms contributing to this neuronal diversification have only been partly deciphered. In this study, we aimed to identify additional markers to further describe the diversity of spinal V2 interneuron populations. Here, we compared the transcriptome of V2 interneurons with that of the other cells of the embryonic spinal cord and extracted a list of genes enriched in V2 interneurons, including *Arid3c*. Arid3c identifies an uncharacterized subset of V2 that partially overlaps with V2c interneurons. These two populations are characterized by the production of Onecut factors and Sox2, suggesting that they could represent a single functional V2 unit. Furthermore, we show that the overexpression or inactivation of *Arid3c* does not alter V2 production, but its absence results in minor defects in locomotor execution, suggesting a possible function in subtle aspects of spinal locomotor circuit formation.

## 1 Introduction

Motor activity is organized by neuronal networks located in the brainstem and in the spinal cord. These circuits are composed of motor neurons (MNs) that directly innervate skeletal muscles and of a wide variety of pre-motor interneuron (IN) populations that ensure a precise regulation of the MN activity (Sengupta and Bagnall, 2023; Wilson and Sweeney, 2023). Although the first cardinal populations generated in the embryonic spinal cord and the mechanisms that regulate their production have been extensively described, differential expression and single-cell RNA sequencing (scRNAseq) studies more recently unveiled that these populations subdivide into multiple subsets (Francius et al., 2013; Bikoff et al., 2016; Gosgnach et al., 2017; Hayashi et al., 2018; Sweeney et al., 2018; Delile et al., 2019; Harris et al., 2019; Russ et al., 2021), some of them containing only a few cells, that can display specific identity, location, connectivity, and functions (Borowska et al., 2013; Borowska et al., 2015; Bikoff et al., 2016; Sweeney et al., 2018; Deska-Gauthier et al., 2024). However, some IN subsets have not been described yet, and the mechanisms contributing to this neuronal diversification in the developing spinal cord have only been partly deciphered.

In the ventral neural tube, different neuronal populations arise from specific progenitor domains orderly distributed along the dorsoventral axis, from pdI6 and p0–p3 domains for the different IN populations and pMN for the MNs (Catela et al., 2015; Lu et al., 2015; Gosgnach et al., 2017; Côté et al., 2018; Sengupta and Bagnall, 2023; Wilson and Sweeney, 2023). The p2 progenitor domain, characterized by the combined expression of *Irx3*, *Nkx6.1*, *Nkx6.2*, and *Pax6*, gives rise to the cardinal V2 IN population (Briscoe et al., 2000; Ulloa and Marti, 2010). During neurogenesis, it first produces an intermediate population of V2 precursors, characterized by the transient expression of the transcriptional repressor *Vsx1* (Francius et al., 2016; Debrulle et al., 2020; Baudouin et al., 2021). As development proceeds, these precursors differentiate into at least five different types of V2 INs: V2a, V2b, V2c, V2d, and V2-Pax6. These V2 IN populations are characterized by the expression of specific markers and by a specific role during motor control. The cardinal V2 population firstly divides into V2a INs expressing *Chx10* and representing 35% of all V2 INs at embryonic day 14.5, namely when the production of ventral INs is completed (Sagner and Briscoe, 2019), and into V2b INs expressing *Gata3* and representing 25% of all V2 INs, upon the regulation of the Notch signaling pathway (Li et al., 2005; Del Barrio et al., 2007; Kimura et al., 2008; Misra et al., 2014). V2a INs contribute to the regulation of the left–right alternation during high-speed locomotion (Crone et al., 2008), while V2b INs regulate the alternation of contraction of flexor and extensor muscles (Zhang et al., 2014; Britz et al., 2015). Second, additional V2 subsets arise from those two initial V2 populations. V2d INs expressing *Shox2* derive from V2a INs and participate in the generation of the locomotor rhythm (Dougherty et al., 2013). V2c INs, characterized by the expression of *Sox1*, are reported to derive from V2b but adopt their own identity and migrate ventrally near the MNs (Panayi et al., 2010). In zebrafish, similar cells called V2s INs are inhibitory glycinergic neurons and appear to be involved in rapid motor responses (Gerber et al., 2019). In mice, their involvement in locomotion has not been determined. Finally, from V2b INs arise a late V2 IN population, V2-Pax6, characterized by the expression of *Pax6* (Panayiotou et al., 2013). Their function in motor control has not been reported. These last three populations only represent approximately 10% of V2 INs (Li et al., 2010), which suggests that all the V2 IN subpopulations have not been characterized yet. To address this question, we previously generated a *Vsx1-CreER^T2^* mouse line, enabling us to perform a lineage tracing of all the V2 INs. Analyses of this lineage tracing unveiled the existence of a dorsal Sox1+ V2 IN population that represents ~10% of total V2 INs but confirmed that approximately 25% of V2 INs remain to be described (Baudouin et al., 2021). Identification of specific markers for these populations would enable to characterize their identity and function.

AT-rich interacting domain 3c (Arid3c) is part of the Arid3 family of transcription factors, which also includes Arid3a and Arid3b. These regulators act mainly as transcriptional activators (Herrscher et al., 1995; Numata et al., 1999; Tidwell et al., 2011; Saadat et al., 2021), although a transcriptional repressor activity has been reported in specific contexts (Wilsker et al., 2002). They are characterized by two functional domains that play crucial roles in their functions. On the one hand, the Arid domain is a helix-turn-helix motif-based DNA-binding domain (Wilsker et al., 2002; Patsialou et al., 2005). On the other hand, the REKLES domain, divided into a moderately conserved N-terminal REKLES-*α* region and a highly conserved C-terminal REKLES-*β* domain, is involved in homo- or heteromerization, in nuclear import and export and in association of the Arid3 factors with the nuclear matrix (Kim et al., 2007; Tidwell et al., 2011). In adults, Arid3a is mainly produced by B lymphocytes but is more broadly expressed during embryogenesis, especially in the early lymphoid progenitors (Herrscher et al., 1995; Wilsker et al., 2002; Webb et al., 2011; Ratliff et al., 2014). It regulates gene expression programs and alters differentiation and maintenance of cellular identity in a variety of tissues, particularly in the hematopoietic system (An et al., 2010; Ratliff et al., 2014; Kurkewich et al., 2016; Ward et al., 2016; Rhee et al., 2017; Herrscher et al., 1995). Arid3b is mainly expressed in the testes and leukocytes, but its expression can be detected in a wide variety of other organs such as the thyroid, prostate, and thymus (Wilsker et al., 2002; Takebe et al., 2006; Samyesudhas et al., 2014). It is required for proper cardiovascular and craniofacial development (Takebe et al., 2006; Casanova et al., 2011; Uribe et al., 2014) and B lymphocyte differentiation (Kurkewich et al., 2016) and can bind the same matrix-associated region in the regulatory sequences of the immunoglobulin heavy chain genes as Arid3a (Numata et al., 1999). Finally, Arid3c expression is more restricted and has mainly been described in the hematopoietic system, although some data suggest additional expression in the cerebellum and in the brain (Tidwell et al., 2011; Samyesudhas et al., 2014). Arid3c contributes to B lymphocyte differentiation by co-activating the transcription of immunoglobulin heavy chains mediated by Arid3a (Tidwell et al., 2011). Associated with NPM1 for nuclear shuttling through its REKLES-*β* domain, Arid3c directly regulated the transcription of STAT3, STAT1, and JUNB, therefore promoting monocyte-to-macrophage differentiation (Kim et al., 2024). However, in the vertebrate nervous system and more specifically in the spinal cord, the expression and roles of these 3 *Arid3* genes have never been reported, whereas their orthologs in invertebrates regulate some aspects of CNS development, including axonal guidance, neuronal terminal differentiation, or late development of longitudinal glia in *C. elegans* and *Drosophila*, respectively (Shandala et al., 2003; Ditch et al., 2005; Li et al., 2023).

In this study, using a Vsx1-CreER^T2^ lineage tracing of V2 INs (Baudouin et al., 2021), we compared the transcriptome of V2 INs with that of all the other cells of the spinal cord using RNA sequencing. Among the genes enriched in V2 INs, we elected to focus on *Arid3c*. Here, we provide evidence that Arid3c identifies a subset of V2 INs that partly overlaps with the V2c population and is characterized, as V2c INs, by the presence of Sox2 and Onecut transcription factors, suggesting that they may constitute a single functional V2 population. Furthermore, we show that the inactivation of *Arid3c* results in minor defects in locomotor execution, suggesting a possible function in subtle aspects of spinal locomotor circuit formation.

## 2 Materials and methods

### 2.1 Mouse lines

All experiments were performed strictly in accordance with the European Community Council directive of 24 November 1986 (86–609/ECC) and the decree of 20 October 1987 (87–848/EEC). Mice were raised in our animal facilities and treated according to the principles of laboratory animal care, and experiments and mouse housing were approved by the Animal Welfare Committee of Université catholique de Louvain (Permit Numbers: 2017/UCL/MD/008 and 222801). *Vsx1-CreER^T2^* and *Rosa26R-tdTomato* mice have been previously described (Madisen et al., 2010; Baudouin et al., 2021). The *Arid3c^−/−^* null line was generated using CRISPR-Cas9 mediated homologous recombination. Two ultramers-loxP sequences (5’-AAGAGCAGGCTGAGCCATGGAAATACTTCCCTGATTCTCCTCTCCCTGTATTTGCCACGTGCTAGCATAACTTCGTATAATGTATGCTATACGAAGTTATGCAAGGTCTGAGCTACCTAGAGGCACCACTAGCTAGGACAGGTCGCGTAGATAGATTAGA-3′ and 5’-CCTGTCCTGCAGCAGGAGGGAATACAGTTAGACTGAAGGAAGGCTTTCCTGAAGGATGGGGAATTCATAACTTCGTATAATGTATGCTATACGAAGTTATCCGGGGCCTTGTGAATTGGGTAGTGGAGAAGGGATTTCTCCCTAGAAAATTTCAGTATTG-3′), two Arid3c-crRNA (5’-GGTAGCTCAGACCTTGCACG-3′ and 5’-CTTTCCTGAAGGATGGGCCG-3′) and Cas9 were injected in male pronuclei to target the exon 2 of the Arid3c gene. This enabled the simultaneous obtention of *Arid3^+/−^* mice and of Arid3c*^+/flox^* conditional mice (Supplementary Figure S1A). The removal of the second exon of *Arid3c* leads to a frameshift in the ORF and the creation of an early STOP codon, which causes a loss of 74% of the C-terminal part of the protein, including the ARID and REKLES domains. The *Arid3c^+/−^* knockout (223 bp) or wild type (961 bp) alleles were identified by PCR using primers 1 and 2: 5’-GGCTCTGAGTGTCCATCCTC-3′ and: 5’-CGGAAGTGATGGACTGCTGC-3′. The *Arid3c^flox^* allele (858 bp) was identified by PCR using primers 3 and 4: 5’-TGTATTTGCCACGTGCTAGCATAACTTCGTATAAT-3′ and 5’-CCCAATTCACAAGGCCCCGGATAACTTCGTATAGC-3′ (Supplementary Figure S1B). Further details are available upon request. *Arid3c^+/−^* mice were then crossed to generate *Arid3c^−/−^* constitutive knockout embryos, using *Arid3c^+/+^* embryos as controls. The Arid3c protein was undetectable in the spinal cord of *Arid3c^−/−^* embryos (Supplementary Figure S1C). The morning of the vaginal plug was considered embryonic day (E)0.5. The embryos were collected at embryonic day E 10.5, 11.5, 12.5, 14.5, 16.5, or 18.5. For crossings with *Vsx1-CreER^T2^* transgenic mice, pregnant females were injected intraperitoneally with tamoxifen (100 mg/kg) twice at E9.5 to activate CreER^T2^ activity.

### 2.2 FACS, RNA purification, and RNA-sequencing

Six spinal cords from E14.5 *Vsx1|tdTomato* embryos were dissected and dissociated using a neural tissue dissociation kit (MACS; Miltenyi Biotec #130-092-628) according to the manufacturer’s instructions. Dissociated cells were sorted by FACS (BD FACSAria III) to separate tdTomato^+^ cells (V2 INs) from tdTomato- cells (all the other cells of the spinal cord). Sorted cells were collected in TRIzol reagent, and RNA was purified with the RNeasy micro kit (QIAGEN #74004). RNA concentration and quality were assessed using a Bioanalyzer (Agilent) and submitted to Genewiz to prepare an ultra-low input RNA-seq library before sequencing with an Illumina HiSeq. Preliminary data were analyzed by Genewiz using the standard RNA-seq data analysis package.

### 2.3 scRNAseq re-analysis

The raw data were already normalized and were further log2 transformed with a pseudo count of 1. Since the focus of the analysis was to compare cells expressing *Arid3c* versus cells expressing *Gata3* or *Chx10*, the 33 clusters grouping from Delile et al. (2019) were subjected to filtering with the following rules: a cluster was included if it contained at least either 5 cells expressing *Arid3c*, or 10 cells expressing *Gata3* or *Chx10*. Cells expressing both *Gata3* and *Chx10* were excluded and cells expressing *Arid3c* were grouped as a new additional cluster. Then, pairwise cluster comparisons were performed with the scoreMarkers function from the R (version 4.3.3)^1^ package scran [version 1.30 (Lun et al., 2016)], with a blocking effect of the developmental stage of the cell. The value of the “log Fold Change detected” (logFC.detected) for all the pairwise comparisons of *Arid3c*^+^ cells versus each of the other clusters of cells was measured to rank candidate markers as it measures the log-fold change in the proportion of cells with detected expression between clusters (Amezquita et al., 2020). A positive value indicates that a greater proportion of *Arid3c*^+^ cells express the gene of interest compared to the other cluster. The minimum value of the logFC.detected (min.logFC.detected) across the pairwise comparisons was used to rank candidate markers as it corresponds to the most stringent value to find upregulated genes in the *Arid3c*^+^ cluster versus all of the other clusters (Amezquita et al., 2020).

### 2.4 *In ovo* electroporation

*In ovo* electroporation was performed at Hamburger–Hamilton (HH) stage 15-16 and embryos were collected at HH26 (72 h after electroporation). The electroporated plasmid was pCIG-Arid3c-IRES-EGFP (1 μg/μl). Details about plasmids are available upon request.

### 2.5 *In situ* hybridization

Collected embryos were fixed in ice-cold PBS/4% paraformaldehyde (PFA) overnight. After washes in PBS, the fixed embryos were incubated in PBS/30% sucrose overnight at 4°C, embedded and frozen in PBS/15% sucrose/7.5% gelatin in isopentane at −55°C. *In situ* hybridization was performed on 14–20 μm serial cryosections. On the first day, slides were post-fixed in PBS/4% paraformaldehyde for 10 min and then acetylated in a 0.1 M triethanolamine–HCl–acetic anhydride (pH8) solution for 10 min. After blocking non-specific hybridization for 2 h at RT in hybridization buffer (50% formamide/10% dextran sulfate/10% salt 10x (NaCl 2 M/Tris–HCl 0.1 M pH7.5/NaH_2_PO_4_, 2H_2_O 50 mM/Na_2_HP O_4_ 20 mM/EDTA 50 mM)/2% Denhardt’s 50x/1 mg/mL of tRNA), slides were incubated with 300 ng of DIG-labeled probe diluted in hybridization buffer overnight at 65°C. The next day, slides were washed four times for 45 min in a 1xSSC/50% formamide/0.1% Tween 20 washing buffer at 65°C and three times for 30 min in a 20% MAB5x (maleic acid 100 mM/NaCl 150 mM pH7.5)/0.1% Tween 20 buffer.

Slides were then subjected to an RNase treatment with 1:2000 diluted RNase T1 (Roche #10109193001) in an RNase buffer (Tris–HCl 10 mM pH7.5/400 mM NaCl/5 mM EDTA) for 30 min at 37°C. After a last 30 min wash in MABT buffer, slides were incubated with a 1:4000 anti-digoxigenin antibody (Roche #11093274910) in a blocking solution (1x MAB/0.1% Tween 20/2% Boehringer reagent/20% Horse serum) overnight at 4°C after a blocking step of 1 h in the same solution. On the last day, slides were washed three times for 30 min in MABT buffer and two times for 15 min in an alkaline phosphatase buffer (100 mM Tris–HCl pH9.5/100 mM NaCl/50 mM MgCl_2_/0.1% Tween 2). For the detection of the probe, slides were incubated with 1:2000 diluted NBT (Roche #11383213001) and 1:350 diluted BCIP (Roche #11383221001) in alkaline phosphatase buffer. This solution was changed every 2 h until the staining was complete. Finally, the staining was stopped by washes in 1x PBS/0.1% Triton. Slides were washed in 1x PBS and mounted with Dako Fluorescence Mounting Medium (Dako #S3023). The probes used were DIG-conjugated *Arid3c* antisense (primer pair: 5′-CCCCAAGAGGAAGGAGTTTC-3′ and 5′-GCGTCGAGCAAAGAGGATAC-3′), *Arid3a* (primer pair: 5′-CTGTCTTAGCCGCACAATCA-3′ and 5′-AGGGTGACCAGGAAGGTTCT-3′) and *Arid3b* [primer pair: 5′-CAAGAACCCAGAGCAACACC-3′ and 5′-GAGTAGAGCCCGCAATGAGG-3′(Casanova et al., 2011)] RNA probes.

### 2.6 Immunofluorescence labeling

Collected embryos were fixed in ice-cold PBS/4% paraformaldehyde (PFA) for 15–25 min, depending on the developmental stage. After washes in PBS, the fixed embryos were incubated in PBS/30% sucrose overnight at 4°C, embedded and frozen in PBS/15% sucrose/7.5% gelatin in isopentane at −55°C. Immunolabeling was performed on 14 μm serial cryosections as previously described (Espana and Clotman, 2012). Primary antibodies against the following proteins were used: rabbit anti-Arid3c (Tidwell et al., 2011) at 1:1000, sheep anti-Chx10 (Exalpha Biologicals #X1179P) at 1:200, goat anti-Foxp1 (R&D #AF4534) at 1:500, rat anti-Gata3 (Absea Biotechnology #111214D02) at 1:15 or rabbit anti-Gata3 (Cell signaling #5852) at 1:200, chicken anti-GFP (Aves lab #GFP-1020) at 1:2000, guinea pig anti-OC1/HNF6 (Espana and Clotman, 2012) at 1:6000, goat anti-Isl1 (R&D #AF1837) at 1:1000, rat anti-OC2 (Clotman et al., 2005) at 1:40, guinea pig anti-OC3 (Pierreux et al., 2004) at 1:6000, goat anti-Sox1 (R&D # AF3369) at 1:500 and mouse anti-Sox2 (Invitrogen # 20G5) at 1:250. Following secondary antibodies were used: donkey anti-chicken/Alexa Fluor 488, donkey anti-goat/Alexa Fluor 594, donkey anti-goat/Alexa Fluor 488, donkey anti-goat/Alexa Fluor 647, donkey anti-mouse/Alexa Fluor 594, donkey anti-mouse/Alexa Fluor 488, donkey anti-rabbit/Alexa Fluor 594, donkey anti-rabbit/Alexa Fluor 488, donkey anti-rabbit/Alexa Fluor 647, donkey anti-rat/Alexa Fluor 594, donkey anti-rat/Alexa Fluor 488, donkey anti-guinea pig/Alexa fluor 488 purchased from Thermo Fisher Scientific or Jackson Laboratories and used at dilution 1:500.

### 2.7 Imaging

Immunofluorescence or *in situ* hybridization images of cryosections were acquired on an epifluorescence microscope EVOS FL Auto Imaging System (Thermo Fisher Scientific), on a Zeiss AXIO Observer Z1 Inverted LED Fluorescence Motorized Microscope using ZEN Blue Zeiss software, on a Leica DM2500 microscope with a Leica DFC420C camera, on a confocal laser Scanning biological microscope FV1000 Fluoview with the FV10-ASW 01.02 software (Olympus) or on a confocal microscope Leica Stellaris 8 Falcon using Las X software. The images were treated with Fiji-ImageJ, Adobe Photoshop CC, or Las X office software to match the brightness and contrast with the observations.

### 2.8 Motor behavior tests in adult mice

Motor behavior tests were performed on *Arid3c^+/+^* control mice (two males and nine females) or *Arid3c^−/−^* null mice (four males and five females) at approximately 2 months of age. All mice had a week of acclimation before starting experiments and were weighed each day. For physiocage testing, each animal was housed for 48 h in a metabolic cage in which its motor activity (horizontal and rearing) and its food and water intake were measured by weight or infrared sensors. For the open-field test, each animal was placed in a new environment (Plexiglass box, 60 cm square), and its general activity and motor behavior were analyzed for 20 min. The grip strength test, balance beam test, rotarod, and catwalk test were performed as previously described (Audouard et al., 2012).

### 2.9 Experimental design and statistical analyses

For the quantifications of *in ovo* electroporation experiments, labeled cells were counted on 8–12 sections on each side of the spinal cord in three independent embryos using the count analysis tool of Adobe Photoshop CC software. Quantifications on the “electroporated” (targeted) side of the spinal cord were compared to quantifications on the “non-electroporated” (contralateral) side of the same embryo. For quantifications in mouse embryos, the different levels of the spinal cord (brachial, thoracic, and lumbar) were determined using immunolabeling for Foxp1, which enables the visualization of the lateral motor columns in brachial or lumbar regions. At E12.5, labeled cells were counted on both sides of the spinal cord on 4–11 sections at the brachial level, 6–12 sections at the thoracic level, and 6–13 sections at the lumbar level for each of the 5–6 embryos analyzed per genotype using the count analysis tool of Fiji-ImageJ software. At E14.5, labeled cells were counted on both sides of the spinal cord on 4–11 sections at the brachial level, 3–18 sections at the thoracic level, and 4–10 sections at the lumbar level for each of the 3–6 embryos analyzed per genotype using the count analysis tool of Fiji-ImageJ software. Quantifications in the *Arid3c^−/−^* null mutant embryos were compared with quantifications of *Arid3c^+/+^* control embryos from the same litters. Statistical analyses and graph production were conducted using JMP Pro 17 software. For mouse embryo analyses, differences in cell numbers between two different groups were evaluated using either a Wilcoxon–Mann–Whitney’s non-parametrical test, a Welch’s *t*-test, or a Student’s *t*-test, depending on the normality and the homoscedasticity of the data. For motor behavior analyses, tests were performed on 9 *Arid3c^−/−^* null mice (4 males, 5 females) and 11 *Arid3c^+/+^* control mice (2 males, 9 females). Statistical analyses and graph production were conducted using JMP Pro 17 Software. For the open-field, physiocage, balance beam, rotarod, grip strength test, and catwalk, differences between the two experimental groups were evaluated using either a Wilcoxon–Mann–Whitney’s non-parametrical test, a Welch’s *t*-test, or a Student’s *t*-test, depending on the normality and the homoscedasticity of the data. For the catwalk test, the calculated *p*-values were adjusted using the Benjamini–Hochberg *p*-value correction, and some parameters (print maximum area, print intensity, print width, and print length) were adjusted according to the weight of each animal in order to avoid any weight-related bias. In all statistical analyses, a *p* < 0.05 was defined as significant.

## 3 Results

### 3.1 Arid3c is enriched in V2 INs during spinal cord development

**Figure 1:**
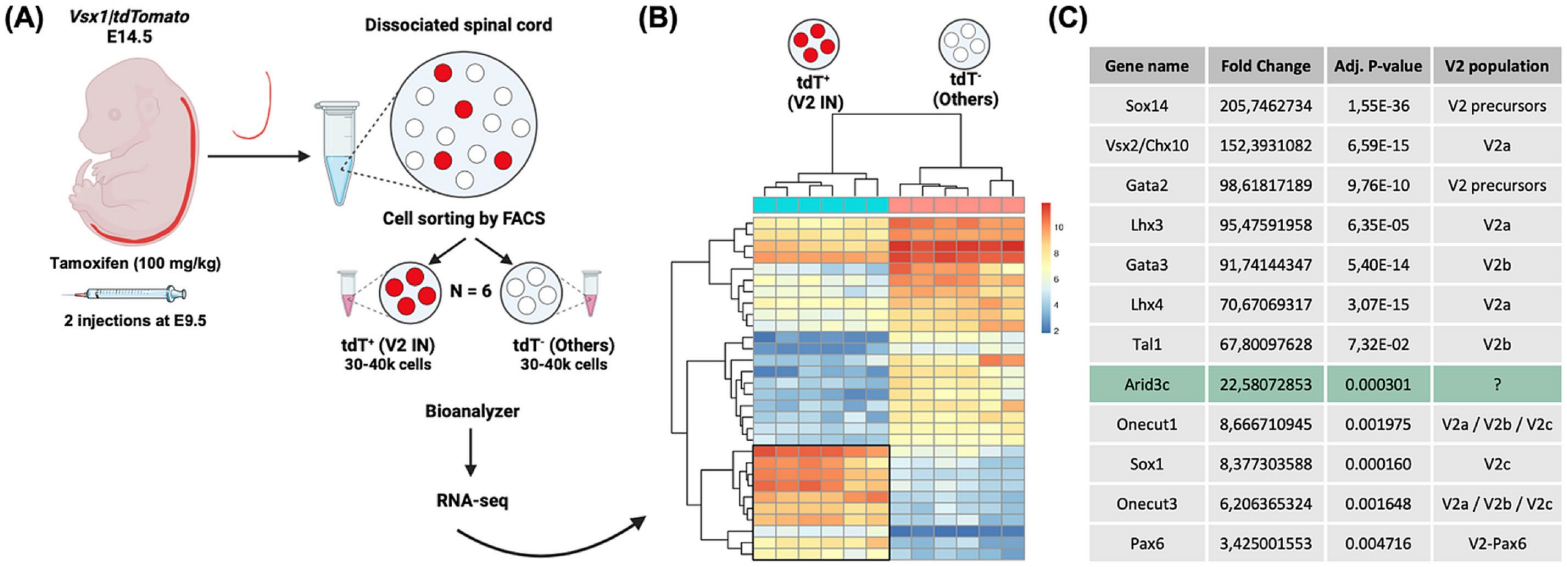
Identification of genes enriched in V2 interneurons (INs) during spinal cord development. **(A)** Schematic representation of the experimental design for RNA-seq experiment on V2 INs. Spinal cords of *Vsx1|tdTomato* embryos were collected at embryonic day (E)14.5 from females injected twice with tamoxifen (100 mg/kg) at E9.5 and were dissected and dissociated. FACS sorting of tdTomato^+^ cells enabled the separation of V2 INs from the other cells of the spinal cord. **(B)** Heat map showing differentially expressed genes across two comparisons (tdT^+^ versus tdT^−^) from each spinal cord sample (*n* = 6). Each row represents the transformed z-score of one differentially expressed gene across all samples (blue, low expression; red, high expression). **(C)** Fold-change comparison between V2 INs (tdTomato^+^ cells) and all the other cells of the spinal cord for known V2 IN markers and for a new candidate gene, *Arid3c*.

Given that our and other studies evidenced that approximately 25% of V2 INs remain to be characterized (Li et al., 2010; Baudouin et al., 2021), we performed an RNA-seq experiment to identify genes enriched in V2 INs during spinal cord development using embryos wherein tdTomato is specifically produced in all the V2 populations [*Vsx1|tdTomato* embryos; Figure 1 (Baudouin et al., 2021)]. Spinal cords of six E14.5 *Vsx1|tdTomato* embryos were dissected and dissociated. Dissociated cells were sorted by FACS to separate the tdTomato^+^ V2 INs from the tdTomato- cells to compare the transcriptome of V2 INs with that of all the other cells of the spinal cord, thereby identifying V2-enriched transcripts (Figures 1A,B). The sequencing results showed a significant enrichment in known V2 markers, including markers of V2 precursors (Sox14, Gata2) (Zhou et al., 2000; Karunaratne et al., 2002; Francius et al., 2014; Clovis et al., 2016), of V2a (Vsx2/Chx10, Lhx3 and Lhx4) (Ericson et al., 1997; Briscoe et al., 2000; Renaux et al., 2024) and of V2b (Gata3, Tal1, also known as SCL) (Karunaratne et al., 2002; Smith et al., 2002; Muroyama et al., 2005) INs with a fold-change superior to 67.8 (adj *p* ≤ 0.0001), which confirms their relevance (Figure 1C). Interestingly, we could also identify genes expressed in very small V2 IN populations such as Sox1 (V2c INs and dorsal V2 population) (Panayi et al., 2010; Baudouin et al., 2021) and Pax6 (V2-Pax6 INs) (Panayiotou et al., 2013), or present in V2 subpopulations like Onecut factors (Francius et al., 2013; Harris et al., 2019), which further validates our analysis (Figure 1C). Here, we decided to focus our study on *Arid3c*, which displayed a 22.6-fold enrichment in V2 INs versus the other cells of the spinal cord (Figure 1C, adj. *p*-value of 0.000301), and which had never been described in V2 INs yet.

### 3.2 *Arid3c* is expressed exclusively in V2 INs, while *Arid3a* and *Arid3b* are not

**Figure 2:**
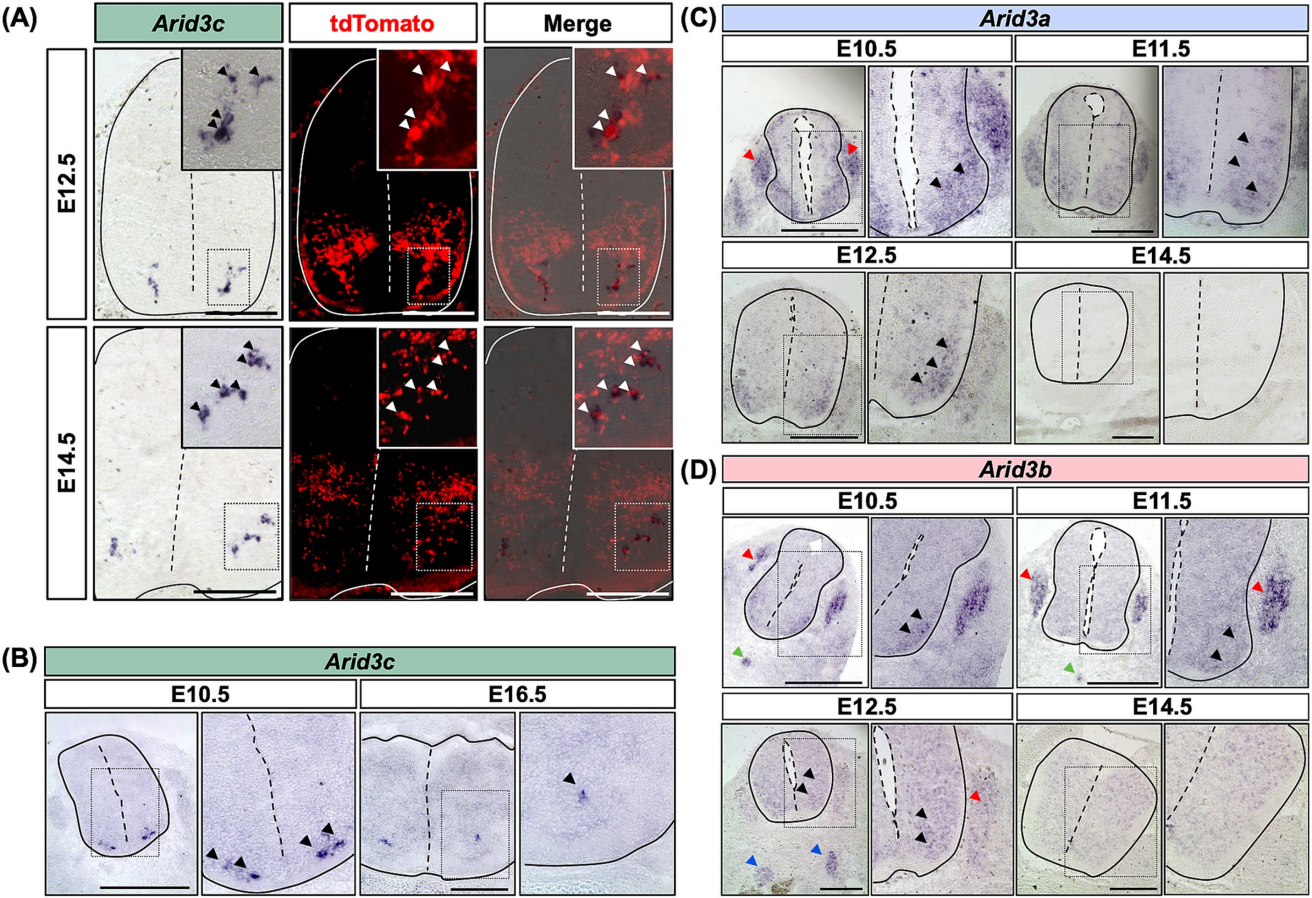
*Arid3c* is expressed specifically in V2 INs, while *Arid3a* and *Arid3b* are not. **(A)** *In situ* hybridization for *Arid3c* on transverse cryosections of the spinal cord of E12.5 or E14.5 *Vsx1|tdTomato* embryos, wherein V2 INs produce tdTomato. Cells expressing *Arid3c* are located in a ventrolateral position close to the motor columns. Overlay with the tdTomato fluorescence demonstrates specific expression in V2 INs, in the ventral-most population that affixes on the medial boundaries of the motor columns. **(B)** *Arid3c* expression is detected by *in situ* hybridization from E10.5 to E16.5 in a similar location, although it is progressively more restricted at later stages. **(C)** *Arid3a* expression is detected by *in situ* hybridization in the ventral part of the spinal cord from E10.5 to E12.5. *Arid3a* is broadly expressed in the spinal cord and in the dorsal root ganglia at E10.5 and E11.5 but its expression decreases from E11.5 and becomes undetectable at E14.5. **(D)** *Arid3b* expression is detected by *in situ* hybridization in the ventral part of the spinal cord from E10.5 to E14.5. It is expressed in MNs (black arrowheads), in the dorsal root ganglia (red arrowheads), and in the notochord (green arrowheads) at E10.5 and E11.5. At E12.5, its expression becomes weaker and more diffuse in the spinal cord and the dorsal root ganglia but is detected in the ventral sympathetic chain (blue arrowheads), while it becomes undetectable at E14.5. Scale bars = 400 μm.

**Figure 3:**
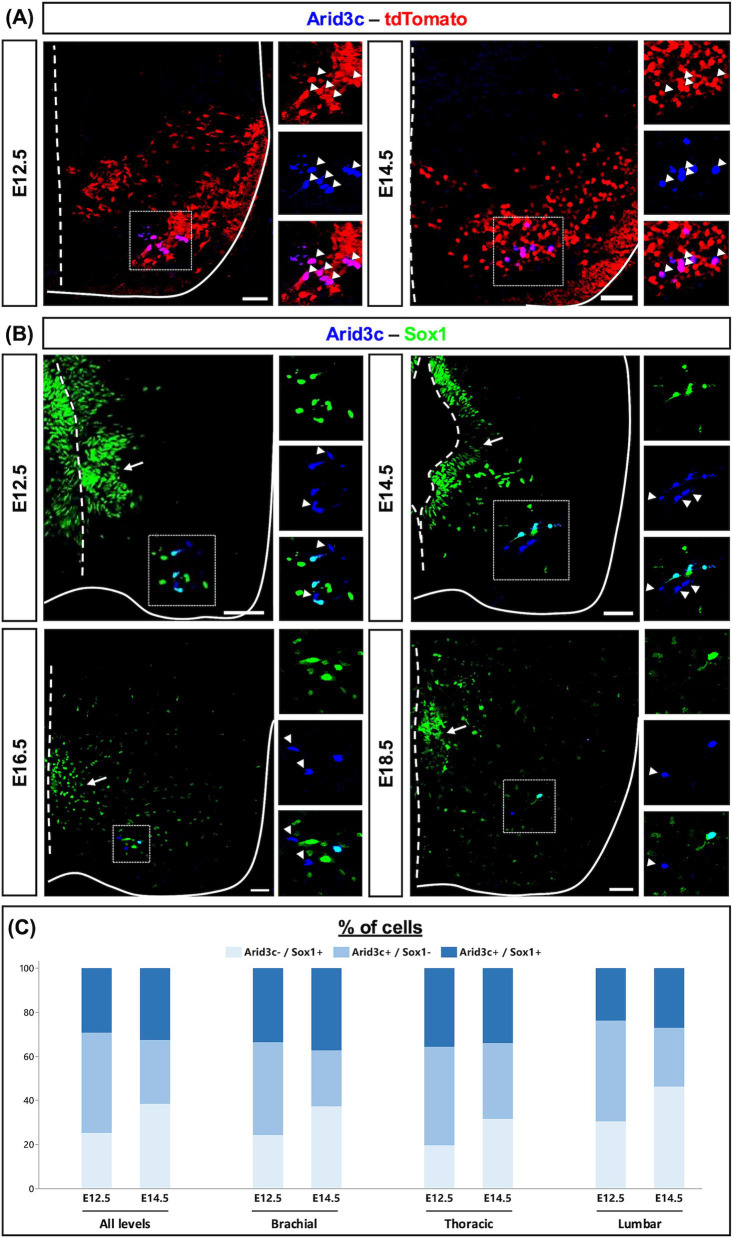
Arid3c is produced in V2c INs and identifies an uncharacterized V2 population. **(A)** Immunolabeling for Arid3c on transverse cryosections of E12.5 or E14.5 *Vsx1|tdTomato* embryos. All the Arid3c+ cells are V2 INs, characterized by the production of tdTomato (arrowheads). **(B)** Immunolabeling for Sox1 and Arid3c on transverse cryosections of E12.5, E14.5, E16.5, or E18.5 embryos or fetuses. Arid3c is detected in some V2c INs, large cells ventrolaterally located and producing high Sox1 levels. Moreover, Arid3c is produced in Sox1^−^ cells located in the vicinity of V2c INs. Arrowheads indicate Arid3c^+^ cells that lack Sox1. Note that Sox1 is also produced in the progenitor cells of the ventricular zone (arrows). **(C)** Quantification of the proportion of cells that contain only Arid3c, only Sox1, or both Sox1 and Arid3c at E12.5 or E14.5 at brachial, thoracic, or lumbar levels of the spinal cord. *n* ≥ 5. Scale bars = 50 μm.

To confirm the potential expression of *Arid3c* in V2 INs, we first performed *in situ* hybridization experiments. Using *Vsx1|tdTomato* embryos at E12.5 or E14.5, *Arid3c* expression could be detected in tdTomato^+^ cells, i.e., V2 INs (Baudouin et al., 2021). *Arid3c* seemed expressed in a ventrolateral subpopulation of V2 INs, located in the vicinity of the motor columns (Figure 2A). *Arid3c* expression could also be observed at E10.5 and E16.5 in the same location, although it seemed more restricted at later stages (Figure 2B). Coimmunofluorescence for Arid3c and tdTomato confirmed that all the Arid3c-producing cells also contained tdTomato, i.e., are V2 INs (Figure 3A). Although this is the first report of *Arid3c* expression in the CNS, published data suggested the expression of its two paralogs, *Arid3a* and *Arid3b*, in the embryonic spinal cord. Therefore, we also assessed the presence of *Arid3a* or *Arid3b* transcripts in this tissue. *Arid3a* was broadly expressed in the developing ventral spinal cord at E10.5 and E11.5 but its expression steadily decreased with time and became undetectable at E14.5, which indicates a more transient expression than *Arid3c* (Figure 2C). *Arid3a* also seemed to be transiently expressed in the dorsal root ganglia at E10.5 and E11.5 (Figure 2C). Regarding *Arid3b*, its expression at E10.5 and E11.5 seemed restricted to a population corresponding to MNs in the ventral spinal cord. It was also detected in the dorsal root ganglia, the notochord, and the presumptive sympathetic chain (Figure 2D). Starting from E12.5, its expression became weaker and more diffuse and was completely undetectable at E14.5 (Figure 2D). As for *Arid3a*, these results indicate a transient and less restricted expression of *Arid3b* than *Arid3c*. Altogether, these results show that the 3 *Arid3* genes are expressed in the embryonic spinal cord, but only *Arid3c* is specific to V2 INs.

### 3.3 Arid3c is produced in some V2c INs and identifies an uncharacterized population of V2 INs

To assess in which V2 population Arid3c is produced, we performed immunofluorescence experiments for Arid3c and different V2 IN population markers. We never observed any colocalization of Arid3c with Chx10 (V2a), Shox2 (V2a/d), or Gata3 (V2b). Furthermore, despite their distribution in the vicinity of the motor columns, Arid3c^+^ cells did not contain Isl1, confirming that these are not MNs (data not shown). In contrast, Arid3c was detected from E12.5 onward in cells containing Sox1 located ventrally and laterally, corresponding to V2c INs (Panayi et al., 2010) (Figure 3B). However, the distribution of those two proteins was not entirely overlapping. Indeed, at E12.5, cells that contained only Arid3c (mean value of 7.2 cells/section, 45.4%), cells showing a co-labeling for Arid3c and Sox1 (4.7 cells/section, 29.3%) and cells producing only Sox1 (4.0 cells/section, 25.2%) could be identified. At E14.5, the proportion of cells containing only Arid3c decreased (2.9 cells/section, 29.0%) at the benefit of Sox1^+^ only cells (3.9 cells/section, 38.4%), while the proportion of Arid3c^+^Sox1^+^ cells remained similar (3.2 cells/section, 32.6%) (Figures 3B,C). At both stages, the proportion between these cell categories was similar between the different levels of the spinal cord (Figure 3C). Similarly, the total number of Arid3c^+^ cells remained constant between the different spinal cord levels, varying between mean values of 9.65, 11.65, and 13.57 cells/section at E12.5 at brachial, thoracic, or lumbar levels respectively, and between 6.88, 5.95, and 6.41 cells/section at E14.5.

This led us to raise three hypotheses regarding the identity of the Arid3c^+^ cells, which could either represent an intermediate differentiation state between V2b and V2c INs, i.e., cells having lost the expression of *Gata3* and transiently expressing *Arid3c* before reactivating *Sox1*, or cells that correspond to another uncharacterized V2 IN population, or complement V2c INs to constitute a single functional V2 subset. To address these hypotheses, we analyzed the colocalization of Arid3c and Sox1 at later developmental stages. Even though the number of cells was very low at E16.5 and E18.5, we were still able to detect the same three cell categories (Figure 3B). In contrast, Arid3c was not detectable in the adult spinal cord (data not shown). Altogether, these results suggest that Arid3c identifies an embryonic population of V2 INs that has not been characterized yet but partly overlaps with the V2c INs characterized by the presence of Sox1. Whether these combined populations constitute a functional subset of V2 INs remains to be investigated.

**Figure 4:**
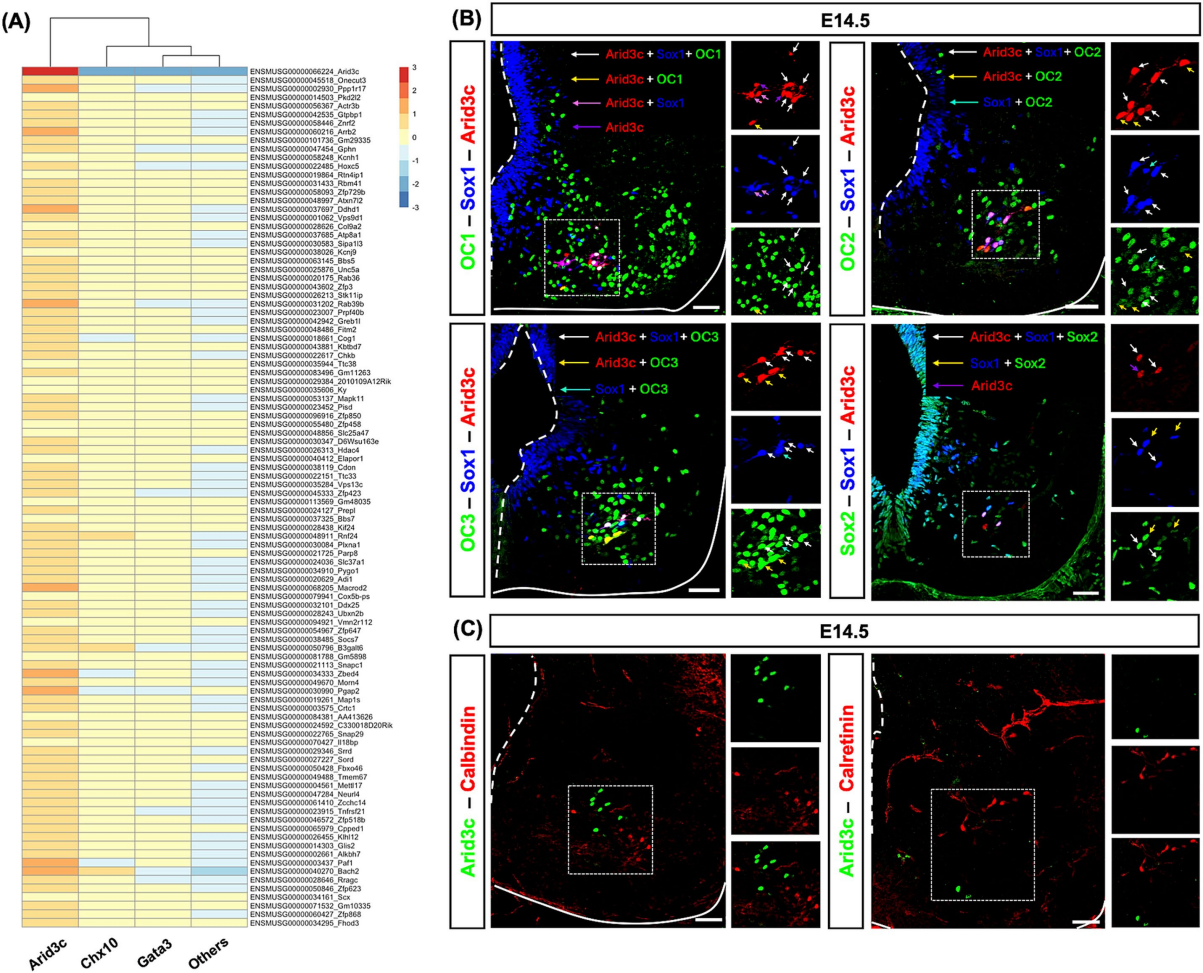
Arid3c^+^ cells display molecular characteristics similar to V2c INs. **(A)** Re-analysis of E9.5–13.5 spinal cord scRNAseq dataset (Delile et al., 2019). Heatmap showing the 100 first genes with the highest min.logFC. detected in Arid3c^+^ clusters as compared to V2a IN (Chx10), V2b IN (Gata3), and the other cells of the spinal cord (others). **(B)** Immunolabeling for Arid3c, Sox1, and OC1, OC2, OC3, or Sox2 on transverse cryosections at E14.5. OC1 is present in a majority of Arid3c^+^ cells or V2c INs (white and yellow arrows), but not all of them (pink and purple arrows), while OC2 and OC3 are produced in all of them (white, yellow, or cyan arrows). Sox2 is detected with Sox1 in V2c INs and in Arid3c^+^ cells. Note that Sox1 and Sox2 are also produced in the progenitor cells of the ventricular zone. **(C)** Immunolabeling for Arid3c and calbindin or calretinin on transverse cryosections at E14.5. Arid3c does not colocalize with calbindin or calretinin. Scale bars = 50 μm.

The diversity of cell populations in the embryonic or postnatal spinal cord has been recently investigated in different scRNAseq studies (Delile et al., 2019; Osseward et al., 2021; Russ et al., 2021). In an attempt to characterize spinal Arid3c^+^ cells, we re-analyzed an embryonic spinal cord scRNAseq dataset (E9.5–13.5) to identify genes that could be specifically co-expressed with *Arid3c* in V2 INs (Delile et al., 2019). V2 subclustering and cluster transcriptome analysis unveiled a list of genes specifically enriched in Arid3c^+^ cells as compared to V2b-Gata3^+^ INs, V2a-Chx10^+^ INs, and the other cells of the spinal cord, although their expression level was lower than that of *Arid3c* (Figure 4A). GO analysis did not significantly associate these genes with specific pathways or biological processes (data not shown). To validate this list of genes and assess if some of these factors could constitute additional markers of this population, we addressed the distribution of corresponding proteins using immunofluorescence. However, none of the tested proteins were exclusively present in Arid3c^+^ cells, although some were enriched in this population. Indeed, a majority of Arid3c^+^ cells and V2c INs were characterized by the presence of OC1, and all of them were producing OC2, OC3, and Sox2 (Figure 4B). Regarding calcium storage proteins, Arid3c^+^ cells were not producing calbindin or calretinin (Figure 4C). Thus, no additional markers of the Arid3c^+^ cells could be identified. However, markers shared with V2c INs added to their similar location reinforce the hypothesis that Arid3c^+^ cells and V2c could represent a single functional V2 population.

### 3.4 Overexpression or loss of Arid3c does not impact the proper production of V2a, V2b, or V2c INs nor the general locomotor behavior

**Figure 5:**
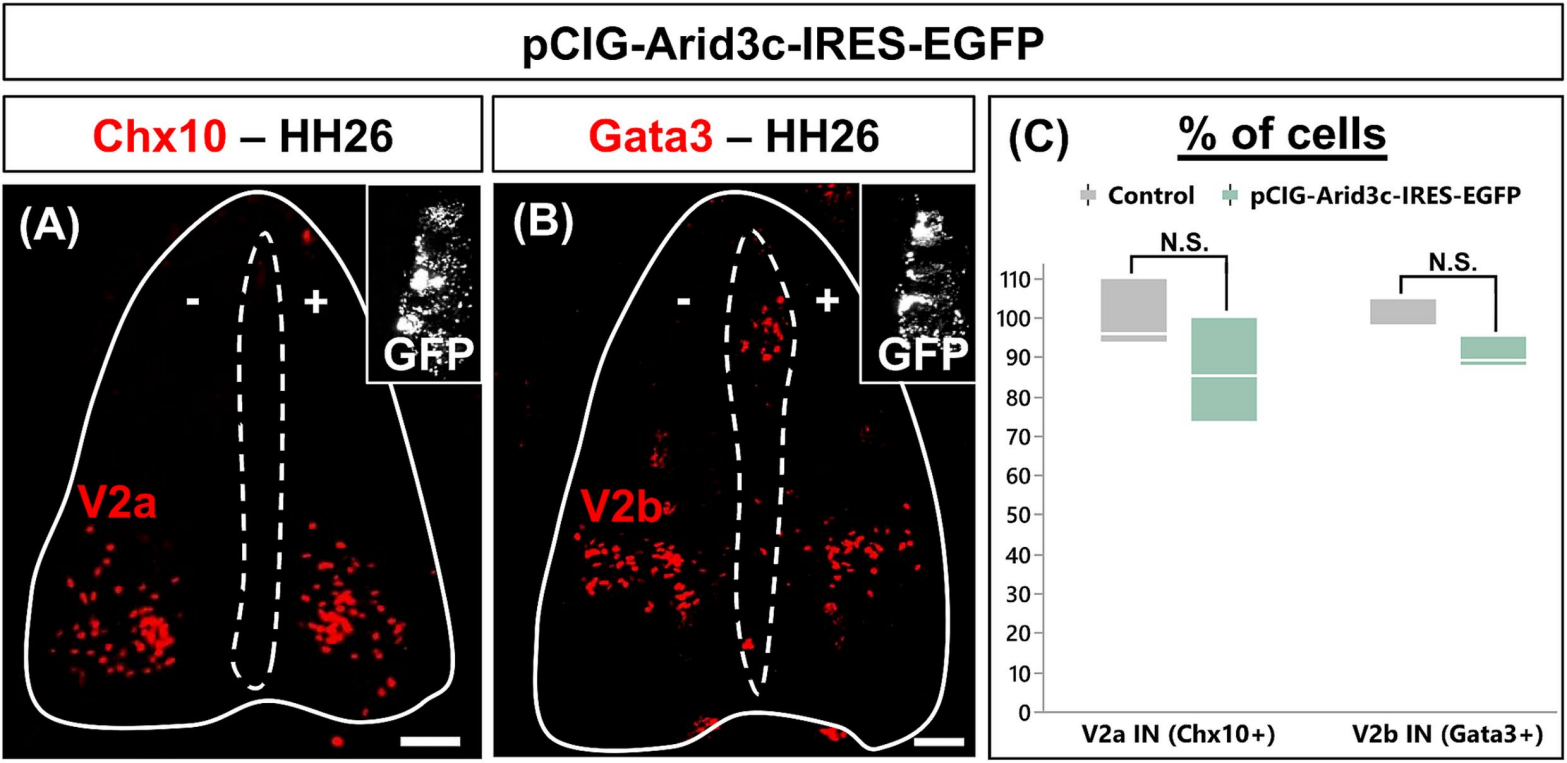
Overexpression of *Arid3c* does not alter V2a or V2b IN production. **(A,B)** Immunolabeling for V2a (Chx10) or V2b (Gata3) INs on transverse cryosections of chicken embryonic spinal cord electroporated with the pCIG-Arid3c-IRES-EGFP expression vector at HH15-16 and collected at HH26. GFP in the insets demonstrates electroporation all along the dorsoventral axis. **(C)** Quantification of the number of V2a or V2b INs on the electroporated side (pCIG-Arid3c-IRES-EGFP, +) of the spinal cord as compared to the control side (−). The overexpression of Arid3c does not significantly impact V2a or V2b IN production. *n* = 3. Scale bars = 50 μm.

Arid3c was detected in part of the V2c INs and in a restricted number of cells located in their direct vicinity, but not in V2a or V2b populations, suggesting that it may stimulate the differentiation of small V2 populations at the expense of the major ones. To test this hypothesis, we analyzed V2 differentiation in HH26 chicken embryos electroporated with an expression vector for *Arid3c*. Ectopic *Arid3c* expression alongside the dorsoventral axis of the spinal cord had no significant impact on the production of V2a or V2b INs (Figure 5; *p* = 0.2071 and 0.0505, respectively). Unfortunately, we could not analyze V2c INs differentiation as we observed that the electroporation process seems to disrupt the organization of some Sox1^+^ progenitors that are displaced laterally from the ventricular zone to the mantle layer, making it impossible to distinguish the Sox1^+^ V2c INs from these artifactually located Sox1 progenitors (data not shown). Nevertheless, these observations indicate that Arid3c is not sufficient to impose an alternative identity on major V2 IN populations.

**Figure 6:**
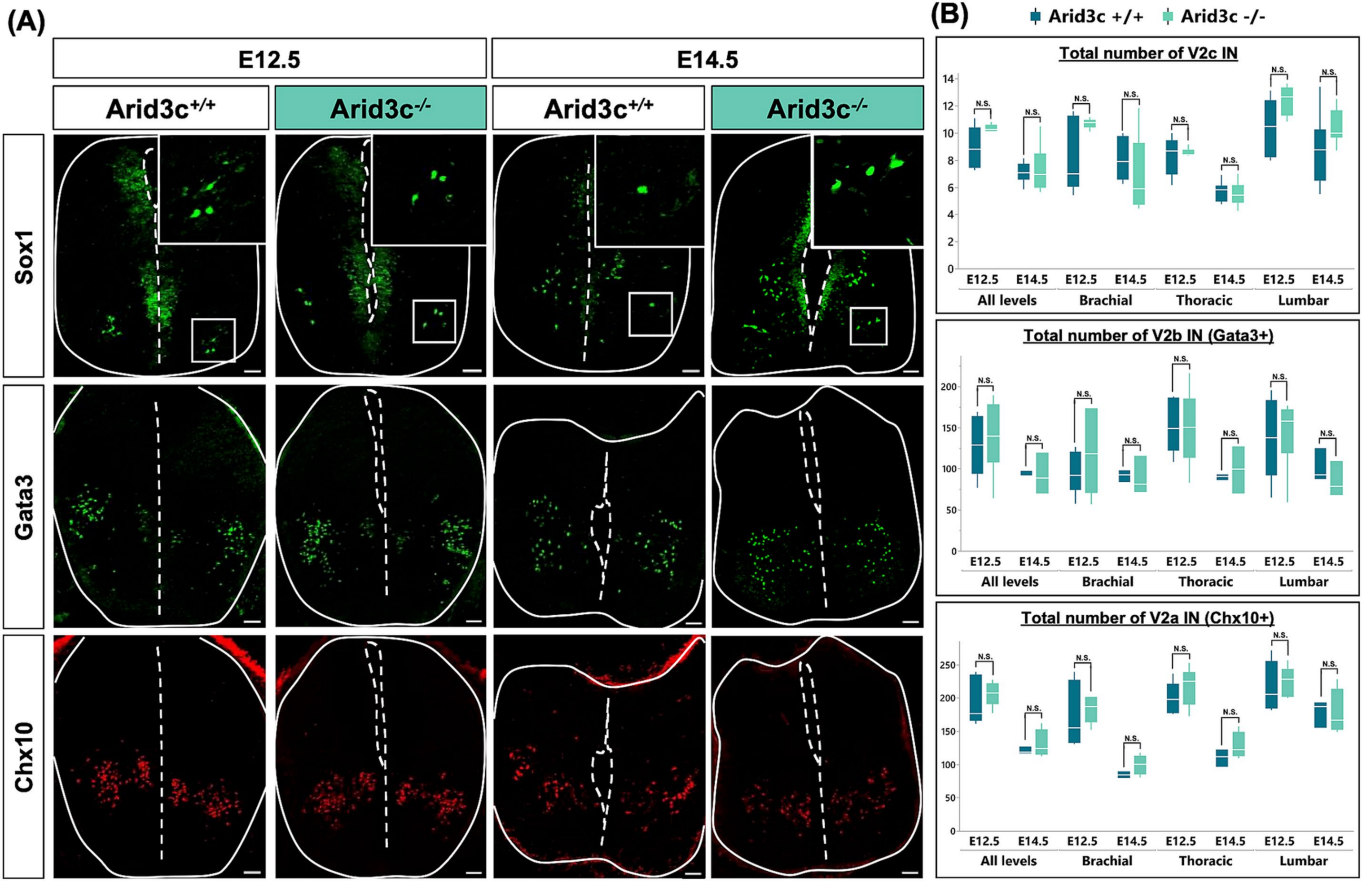
Loss of *Arid3c* does not alter the number of V2a, V2b, or V2c INs. **(A)** Immunolabeling of transverse cryosections of the spinal cord of *Arid3c^+/+^* control or *Arid3c^−/−^* mutant embryos at E12.5 or E14.5. The loss of Arid3c does not alter the number of V2c (ventrolaterally located Sox1^+^ cells), V2b (Gata3), or V2a (Chx10) INs. Note that Sox1 is also produced in the progenitor cells of the ventricular zone. **(B)** Quantification of the number of V2c, V2b, or V2a INs in *Arid3c^+/+^* control or *Arid3c^−/−^* mutant embryos at E12.5 or E14.5 at brachial, thoracic, or lumbar levels of the spinal cord. The loss of Arid3c has no impact on the proper production of V2a, V2b, or V2c INs. *n* ≥ 3. Scale bars = 50 μm.

Alternatively, Arid3c may be necessary for the proper production of smaller V2 IN populations, including V2c (Figures 3, 4). To address the potential roles of Arid3c in V2 IN development and in the formation of the locomotor circuits, we analyzed the phenotype of V2 INs in *Arid3c^−/−^* null mutant mouse embryos (Supplementary Figure S1) at E12.5 and E14.5. The constitutive loss of Arid3c had no impact on the number of V2c INs, whatever the embryonic stage or the level of the spinal cord analyzed (Figures 6A,B). Previous studies suggested that V2c derived from V2b INs (Panayi et al., 2010) and our observations suggest that Arid3c^+^ cells relate to V2c (Figures 3, 4). We assessed whether the loss of Arid3c could impact the amount of V2b INs. However, we could not detect any change in the number of V2b cells regardless of the embryonic stage or the spinal cord level (Figures 6A,B). Consistently, the production of V2a INs was not altered (Figures 6A,B). Taken together, these observations suggest that Arid3c is not necessary for proper production of the V2c INs or for further subdivision of V2b or V2a INs.

**Figure 7:**
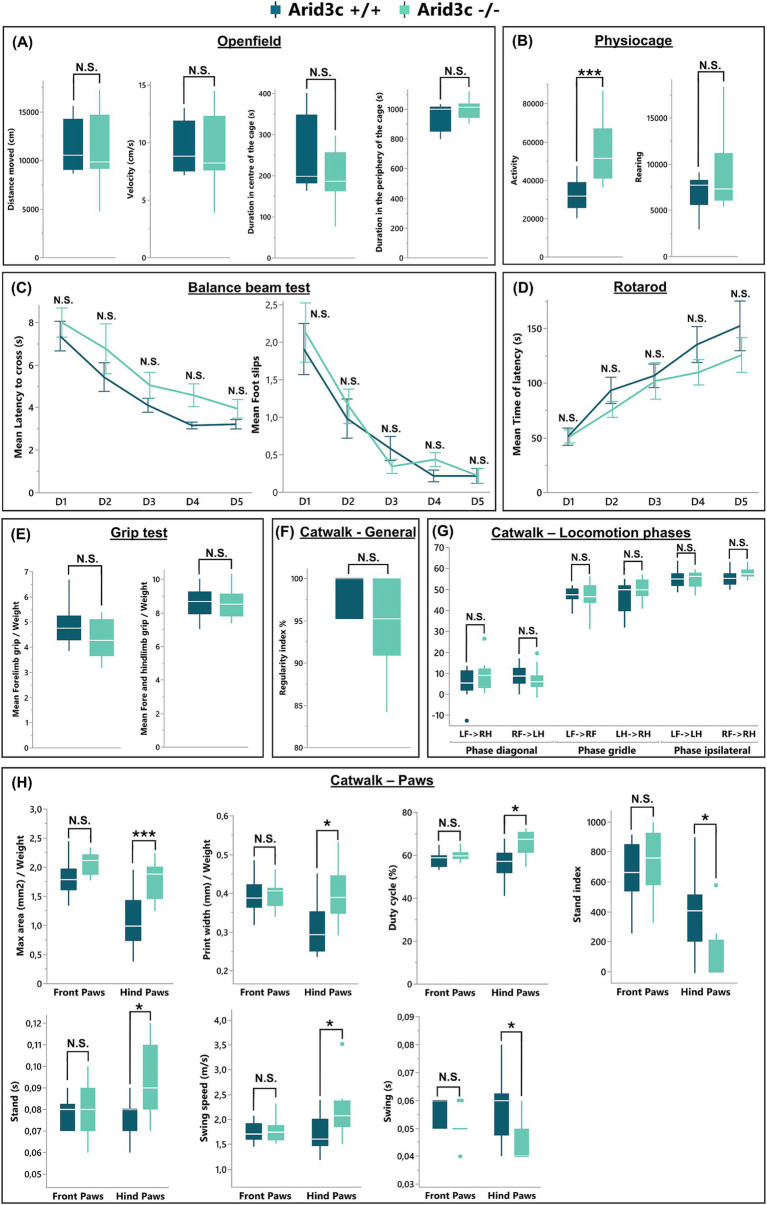
Loss of *Arid3c* has no impact on the general locomotor behavior. Behavioral analysis of general or specific motor functions of *Arid3c^+/+^* control or *Arid3c^−/−^* mutant mice assessed by **(A)** physiocage, **(B)** open-field, **(C)** balance beam, **(D)** rotarod, **(E)** grip strength, or **(F–H)** catwalk tests. **(A)** The loss of Arid3c results in an increase in the general activity of the mice. **(B–G)** Mice lacking Arid3c show no alteration in general locomotion but **(H)** display some minor defects of the hind paw steps. * = adj. *p* < 0.05; *** = adj. *p* < 0.001.

Although the production of the described V2 IN populations was not altered in the absence of Arid3c, later steps of development could be affected, a question difficult to address given the absence of additional specific markers of the V2c and of the Arid3c^+^ INs. To get around this limitation and assess the functional output of the spinal motor circuitry that these cells likely participate in, we characterized the general locomotor behavior of the *Arid3c^−/−^* null mutants. First, a physiocage and an open-field test were performed to analyze the general activity of these mice. Although the loss of Arid3c did not impact any of the parameters analyzed in the open field (total distance, velocity, duration, and frequency in the peripheral or central zones, latency before the first center exploration; Figure 7A and data not shown), the *Arid3c^−/−^* null mice displayed an increased horizontal activity as compared to *Arid3c^+/+^* control mice (Figure 7B; *p* = 0.0008) and a trend to increased rearing (Figure 7B; *p* = 0.0822) that is not explained by an extra intake of food or water (data not shown). However, these observations point to a possible mild anxiety in *Arid3c^−/−^* null mice (Seibenhener and Wooten, 2015), rather than an intrinsic locomotor defect. Consistently, their general motor coordination was comparable to that of *Arid3c^+/+^* control mice in the balance beam (latency to cross the beam and number of foot slips; Figure 7C) or rotarod tests (time of latency before the fall of the animal; Figure 7D), with similar performance improvement with repetitions (Figures 7C,D). As alterations in motor circuit activity can result in reduced muscle strength, this parameter was evaluated in a grip-strength test. However, both *Arid3c^−/−^* null mice and *Arid3c^+/+^* control mice exerted similar forelimb or combined forelimb and hindlimb grip strength (Figure 7E). Finally, possible gait defects of *Arid3c^−/−^* null mice were assessed using a catwalk test. The general locomotion of these mice (regularity index, base of support of the paws, print positions, percentage of support on one, two, three, or four paws) was not altered by the loss of Arid3c (Figure 7F, and data not shown). Consistently, the different locomotion phases that were analyzed (diagonal, girdle, or ipsilateral phases) were similar between mutant or control mice (Figure 7G). However, when looking individually at the front or hind paws, the maximum area and the width of the hind paw prints were increased in *Arid3c^−/−^* null mice (Figure 7H; adj. *p* = 0.0044 and 0.0144, respectively). Similarly, the duty cycle, which expresses stance duration as a percentage of the duration of the step cycle of the hind paws, was higher in *Arid3c^−/−^* null mice (Figure 7H; adj. *p* = 0.0112). Differences in the stand index (measure for the speed at which the paw loses contact with the glass plate), stand (duration of contact of a paw with the glass plate in a step cycle), swing (duration of absence of contact with the glass plate in a step cycle), and swing speed (speed of the paw during swing) of the hind paws were also observed in mice lacking Arid3c (Figure 7H; adj. *p* = 0.0112, 0.0196, 0.0112, 0.0208, 0.04438, respectively). Altogether, those results indicate that even though the general motor behavior of the mice was not impacted by the loss of Arid3c, some subtle aspects of the locomotor execution seem to be altered.

## 4 Discussion

Unraveling the complexity of neuronal diversity and its relationship to CNS functions is a great challenge. Although neuronal populations can be partitioned into multiple subsets based on the specific combination of cell markers, how far does this fragmentation remain biologically significant and which level of granulation corresponds to functional units remains unclear. Here, in an attempt to complete the repertoire of V2 IN populations in the developing spinal cord, we identified a list of genes enriched in V2 INs, including *Arid3c*. We showed that Arid3c identifies a subset of V2 INs that was not characterized yet, whereas *Arid3a* and *Arid3b* are more broadly and transiently expressed in the ventral spinal cord. We observed that this Arid3c^+^ subset partially overlaps with V2c INs and is maintained during development. Moreover, we demonstrated that these cells as well as V2c INs are characterized by the presence of the Onecut factors and of Sox2, suggesting that these two overlapping subsets may constitute a functional unit. However, Arid3c seems to be dispensable for proper production of the V2 IN populations and for the general control of locomotion.

The Arid3 transcription factor family displays a high sequence conservation in their Arid DNA-binding domain (~75%) and in their REKLES-*β* domain (approximately 60%), while the REKLES-*α* region is less conserved (Tidwell et al., 2011). These factors are expressed in the same tissues, such as the hemopoietic and lymphopoietic systems (Herrscher et al., 1995; Wilsker et al., 2002; Tidwell et al., 2011; Webb et al., 2011; Ratliff et al., 2014), and exert partly identical functions, such as the regulation of the expression of the immunoglobulin heavy chain genes (Herrscher et al., 1995; Numata et al., 1999; Kim and Tucker, 2006; Tidwell et al., 2011) or of the cell cycle (Numata et al., 1999; Arman et al., 2020; Saadat et al., 2021). Moreover, the Arid3 proteins are able to collaborate and associate via their REKLES domain in order to achieve those functions (Kim and Tucker, 2006; Tidwell et al., 2011). Accordingly, we detected transcripts of the 3 *Arid3* genes in the developing spinal cord. However, expression of *Arid3a* and *Arid3b* is transient at early stages of neuronal differentiation and is not as restricted as that of *Arid3c*, which is expressed only in V2 INs. This suggested a possible specific role of Arid3c in a V2 population that remained to be characterized, without excluding cooperation of the Arid3 factors at early stages of spinal development.

V2c INs are described to derive from V2b INs, or from cells transiently activating expression from the Gata3 locus, before becoming a distinct and separate neuronal population (Panayi et al., 2010). Those cells then migrate ventrally to finally locate in the vicinity of the MNs. Sox1 is involved in V2c IN differentiation and segregation from V2b cells, as its inactivation results in a reduction in the number of V2c and ectopic expression or maintenance of Gata3 (Panayi et al., 2010), but the mechanisms regulating this segregation remain poorly understood. Similarly, the role of this V2 population has not been elucidated in mice, even though in zebrafish V2s INs that very likely constitute the V2c counterpart are involved in rapid motor responses (Gerber et al., 2019). V2b INs are GABAergic and glycinergic neurons (Zhang et al., 2014). The nature of V2c INs has never been described but zebrafish V2s INs are glycinergic (Gerber et al., 2019; Chen et al., 2023), suggesting that V2c INs may also be inhibitory neurons. Here, we showed that the Arid3c^+^ V2 population partially overlaps with V2c INs. Whether those cells are inhibitory could not be assessed because of the impossibility of obtaining immunofluorescence labeling for Arid3c after *in situ* hybridization (data not shown), and the re-analysis of scRNAseq data did not provide additional indication (data not shown), probably because of the earliness of the embryonic stages analyzed in this study (Delile et al., 2019). The partial overlap between V2c INs and Arid3c^+^ cells could indicate that Arid3c identifies cells in a transition state of differentiation between V2b and V2c INs or a V2 population that may complement V2c. As Arid3c^+^Sox1- cells are maintained at later developmental stages, long after the completion of spinal neurogenesis, our data rather suggest that Arid3c identifies a definitive population of V2 INs. However, they could be part of a larger functional V2 IN unit, including Sox1^+^ cells and Arid3c^+^ cells, functionally independent from the other described V2 populations. Indeed, those two populations share identical characteristics, including the most ventral location for V2 INs and the common expression of *Sox2* and *Onecut* factors. However, studies previously demonstrated that small populations arising from common precursors can acquire molecularly and functionally distinct phenotypes. For example, the small V0_C_ and V0_G_ IN populations both arise from *Pitx2*-expressing cells but are molecularly and functionally different (Zagoraiou et al., 2009; Siembab et al., 2010). Therefore, we cannot exclude that Arid3c^+^ INs, Sox1^+^Arid3c^+^ INs, and V2c (Sox1^+^) INs exert distinct functions, a hypothesis that the tools currently available do not enable to address.

Sox1 is involved in the proper differentiation of V2c INs (Panayi et al., 2010). Therefore, we hypothesized that Arid3c could also be necessary or sufficient to promote V2c fate. However, ectopic Arid3c production in chicken embryonic spinal cord does not reorient the fate of V2a or V2b INs, and the constitutive loss of Arid3c has no impact on the production of a proper number of V2c INs. This does not exclude that Arid3c is implicated in proper differentiation, localization, or function of V2c or Arid3c^+^ INs at later stages of development. Accordingly, the Arid3 ortholog in *Drosophila* is involved in the axonal guidance of specific neuron subtypes, while its *C. elegans* ortholog controls the terminal differentiation of distinct neuronal subtypes, including motor neurons (Ditch et al., 2005; Li et al., 2023). This hypothesis is further supported by alterations of subtle aspects of locomotion in the absence of Arid3c. Unfortunately, the lack of additional markers of V2c or Arid3c^+^ populations currently prevents addressing this question. Furthermore, we do not rule out the possibility of a transient cooperation between Arid3c and its paralog factors, most likely Arid3a, which is also expressed in these two populations at the early stages of their differentiation and may compensate for the absence of Arid3c. Analysis of conditional compound mutants for the Arid3 factors should enable to address this possibility.

## Data Availability

The raw data supporting the conclusions of this article, including RNAseq data, will be made available by the authors, without undue reservation.

## Footnotes

^1^https://eur03.safelinks.protection.outlook.com/?url=https%3A%2F%2Fwww.r-project.org%2F&data=05%7C02%7Cestelle.renaux%40uclouvain.be%7Ccbccc79f7f534befefb408dca2531de9%7C7ab090d4fa2e4ecfbc7c4127b4d582ec%7C1%7C0%7C638563725068922756%7CUnknown%7CTWFpbGZsb3d8eyJWIjoiMC4wLjAwMDAiLCJQIjoiV2luMzIiLCJBTiI6Ik1haWwiLCJXVCI6Mn0%3D%7C0%7C%7C%7C&sdata=XzgKHMeZqhDIu4SH8BST30o2JGaV9WwQhDXw3jeIa6w%3D&reserved=0
