# Supplementary figures and images for: Arid3c identifies an uncharacterized subpopulation of V2 interneurons during embryonic spinal cord development
Source: Front Cell Neurosci. 2024 Oct 16;18:1466056. doi: 10.3389/fncel.2024.1466056 (PMC11521906; doi:10.3389/fncel.2024.1466056)

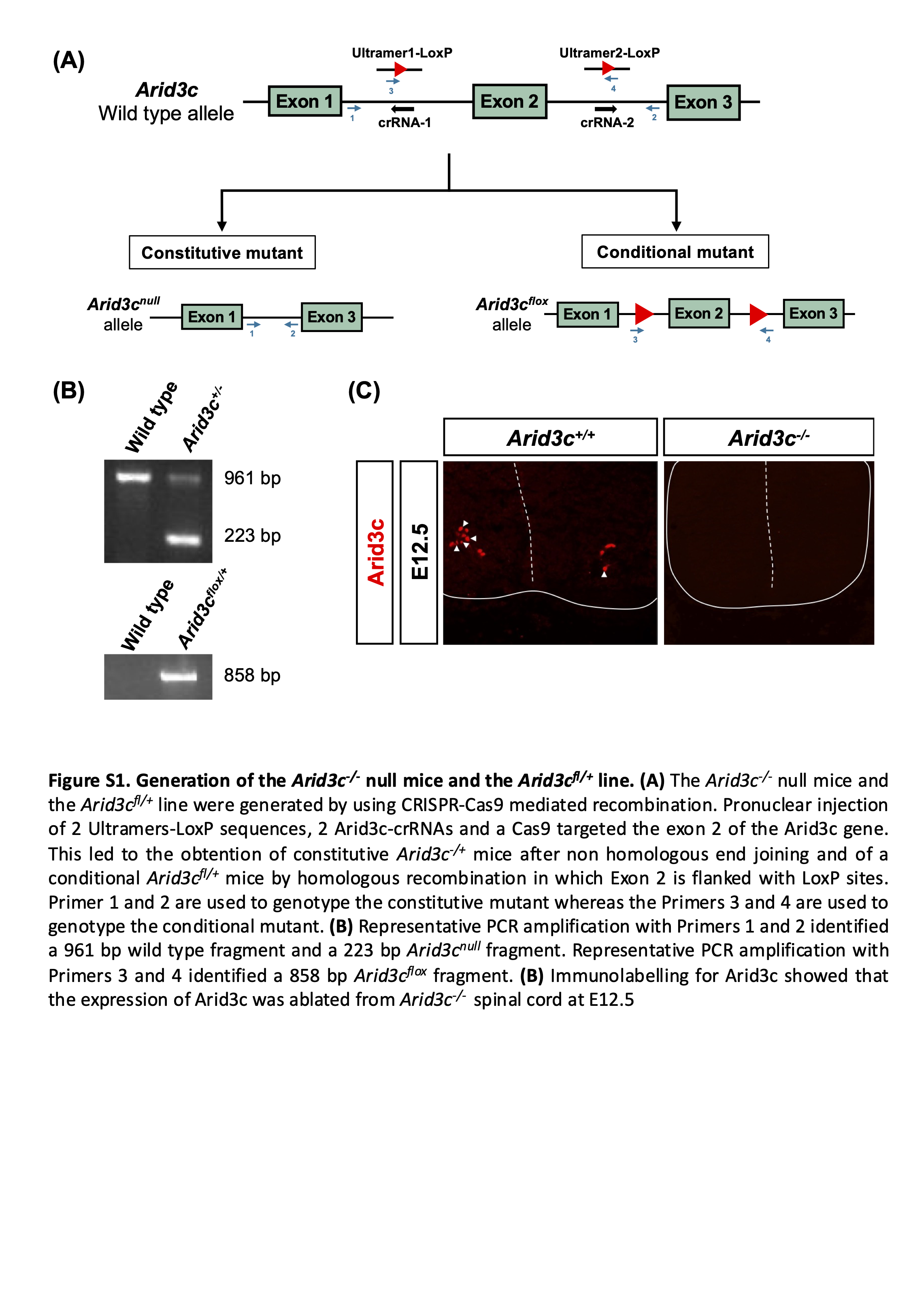

Supplement: Supplementary file 1 [file Image_1.TIFF]
